# Microencapsulation of Tangeretin in a Citrus Pectin Mixture Matrix

**DOI:** 10.3390/foods9091200

**Published:** 2020-08-31

**Authors:** Xiuxiu Sun, Randall G. Cameron, John A. Manthey, Wayne B. Hunter, Jinhe Bai

**Affiliations:** U.S. Horticultural Research Laboratory, Agricultural Research Service, U.S. Department of Agriculture, 2001 S. Rock Road, Ft. Pierce, FL 34945, USA; xiuxiu.sun@usda.gov (X.S.); randall.cameron@usda.gov (R.G.C.); john.manthey@usda.gov (J.A.M.); wayne.hunter@usda.gov (W.B.H.)

**Keywords:** tangeretin, pectin, microencapsulation, natural, spray drying

## Abstract

The objectives of this research were to microencapsulate tangeretin, and to evaluate the basic characteristics of the microcapsule products. Tangeretin is a polymethoxyflavone (PMF) which has been revealed to possess various health benefits and is abundant in tangerine and other citrus peels. Microencapsulation technology is widely employed in the food and pharmaceutical industries to exploit functional ingredients, cells, and enzymes. Spray drying is a frequently applied microencapsulation method because of its low cost and technical requirements. In this research, tangeretin dissolved at different concentrations in bergamot oil was microencapsulated in a citrus pectin/sodium alginate matrix. The resulting microcapsule powder showed promising physical and structural properties. The retention efficiency of tangeretin was greater at a concentration of 2.0% (98.92%) than at 0.2% (71.05%), probably due to the higher temperature of the emulsion during the homogenizing and spray-drying processes. Encapsulation efficiency was reduced with increased concentration of tangeretin. Our results indicate that tangeretin could be successfully encapsulated within a citrus pectin/sodium alginate matrix using bergamot oil as a carrier.

## 1. Introduction

Tangeretin, one of the important polymethoxyflavones (PMFs) obtained from citrus peels, exhibits various functional properties, including being anti-carcinogenic, anti-inflammatory, anti-atherogenic, hepatoprotective, and neuroprotective [1]. Tangeretin has five methoxy groups at positions 4′, 5, 6, 7 and 8 of the flavone skeleton, which bestow upon it a high degree of cell membrane permeability [2]. However, tangeretin is a highly hydrophobic compound with a high melting point, poor water solubility, and a bitter taste, all of which limit its applications for pharmaceutical or food science [3,4]. Therefore, several delivery systems have been developed to overcome these disadvantages. Soy protein isolate was included to enhance the bioaccessibility and physical stability of hydrophobic tangeretin by fabricating supersaturated self-emulsifying nanoemulsion systems, and the combination of 1% soy protein isolate and 50% glycerol obtained a 88% loading efficiency of tangeretin in a stable emulsion system with high transparency [5]. Colloidal delivery systems with tangeretin-loaded protein nanoparticles were successfully developed. These delivery systems were produced by mixing an organic phase containing tangeretin and zein with an aqueous phase containing beta-lactoglobulin, which was then transformed into a powder utilizing a freeze-drying method [6]. Different citrus oils were used in the development of PMF-loaded emulsion-based delivery systems, of which bergamot oil was observed possessing the highest loading capacity and encapsulation efficiency of tangeretin [3]. Among those delivery systems, encapsulation appears to be a promising and effective method [7].

Microencapsulation is a technique in which an active component (often a bioactive ingredient) is enveloped by a protective (often polymeric) layer, on a micro scale [8]. Microencapsulation is a useful tool for improving the efficient delivery of bioactive compounds, such as antioxidants, minerals, and vitamins, into foods [9]. This technology constructs a barrier between degrading environmental conditions and fragile core materials in order to prevent chemical or physical reactions, to alter flavor, to conceal unpleasant aromas or tastes, or to increase the bioavailability of the core material [10]. Different microencapsulation strategies, using nanoparticles [6], emulsion systems [11], and amorphous solid dispersions [12], have been developed to enhance the bioaccessibility of PMFs. Protein-based nanoparticles have been used to encapsulate bioactive tangeretin in order to improve its bioaccessibility [6]. Tangeretin has been encapsulated in highly viscoelastic emulsions in order to achieve high loading efficiency and inhibit crystal sedimentation. Additionally, these emulsions were shown to improve the oral bioavailability and bioactivity of tangeretin and enhance its anticancer efficacy [11]. 

Pectin has ideal properties for incorporation into the microencapsulation process via spray drying, particularly due to its ability to form emulsions even at a relatively low concentration, a characteristic that is crucial for the envelopment of water-repellant components [13,14,15]. Due to its relatively low cost, pectin can be economically used as an emulsifying wall material in the production of microcapsules [16]. The ability of pectin to form gels lends itself to the manufacture of decomposable hydrogels, microbeads, coatings, and films for various microencapsulation applications [17,18]. A biopolymeric blend with pectin and casein was used for the encapsulation of fish oil utilizing a spray-drying method, and this blend showed a higher encapsulation efficiency (64.7–67.9%) and antioxidation efficacy than gum arabic [19]. Multicomponent microcapsules were produced using the precipitation and ionic crosslinking of pectin in a poor solubility environment and with multivalent cations, and the microcapsules stabilized by pectin shells exhibited good structural and functional properties [20]. 

Sodium alginate is a natural gelling agent and negatively charged polyelectrolyte taken from the cell walls of brown algae [8]. Sodium alginate is widely employed for the encapsulation of numerous products, and is used in multiple sectors, such as the food, biomedical, pharmaceutical, and bioprocess industries [13]. The combination of sodium alginate and pectin has been utilized as a wall material capable of producing microcapsules with multiple superior properties than those created with only pectin [13,21]. The combination of pectin and sodium alginate was shown to confer greater capsular stability due to strong synergistic effects under acidic conditions, and therefore microcapsules formed using this combination of components are expected to possess greater encapsulation efficiency due to the increased incorporation and concentration of crosslinked polymers in the final matrix. Alpha-tocopherol encapsulated in a matrix composed of 2.0% (*w*/*v*) pectin and 1.5% (*w*/*v*) sodium alginate was produced with a high encapsulation efficiency [13]. Canthaxanthin was encapsulated in alginate and highly methoxylated pectin through an oil/water/oil multiple emulsion/external gelation method to develop microparticles with improved antioxidant activity [22].

Tangeretin shows high solubility in bergamot oil, especially at temperatures of 60 °C or above [3]. The objective of the present study was the development of an oil-in-water emulsion to obtain microcapsules using sodium alginate and pectin as wall materials, and which are suitable for incorporating tangeretin in to food products.

## 2. Materials and Methods

### 2.1. Materials

Bergamot oil, sodium alginate, methylene dichloride, and Tween^®^ 80 were obtained from Sigma-Aldrich (St. Louis, MO, USA). Sodium sulfate and methanol were acquired from Fisher Scientific (Fair Lawn, NJ, USA). 

### 2.2. Pectin Extraction

*Citrus* × *sinensis* (L.) Osbeck var. Valencia (Spring 2015) oranges were juiced by using a fresh orange juicer (Fresh’n Squeeze^®^; FoodTech, Lakeland, FL, USA), and approximately 200 kg of citrus peel was were collected. The citrus peel waste was stored in bags and buckets and kept at −20 °C. Frozen citrus peel waste was thawed and then processed by using a steam-explosion procedure which integrates high temperature steam conveyance, cooking (softening), and vacuum explosion [23].

For pectin extraction, 100 g of steam-exploded citrus peel waste sludge was suspended in 100 mL of deionized (DI) water containing chloramphenicol (10 mg mL^−1^) and cycloheximide (5 mg mL^−1^) to prevent microbial contamination. The suspended citrus peel waste was shaken for 30 min at 24 °C using a wrist shaker. The suspensions were then centrifuged at 15,000× *g* for 20 min at 4 °C. The pellets were washed three times with DI water and the supernatants were pooled. Pectic hydrocolloids were recovered from the aqueous wash solutions by precipitation with acidified ethanol at 4 °C overnight. The precipitate mixtures were centrifuged at 12,000× *g* for 20 min at 20 °C. After centrifugation the supernatant was discarded and the remaining pellets underwent an acidified ethanol wash and then the centrifugation procedure was repeated. Finally, pellets were submerged in liquid nitrogen and freeze dried (FreeZone Freeze Dry System; Labconco, Kansas City, MO, USA). Freeze-dried pellets were kept at −80 °C. A portion of the freeze-dried pellets were dissolved in water to a final concentration of 1% (*w*/*v*). This material was extensively purified after multiple rinses of DI water using 6000–8000 Da molecular-weight cut-off dialysis tubing (Spectra/Por) overnight. Subsequently, the dissolved pectic hydrocolloids were precipitated and lyophilized as previously described [23], and the final precipitate was used as a wall component for encapsulation.

### 2.3. Tangeretin Extraction

Tangerine oil wax was obtained from winterized tangerine oil, obtained from a local citrus juice-processing plant. The oil was decanted and the remaining solid precipitates were washed repeatedly with hexane to remove volatile oil constituents; primarily limonene, and other hexane soluble components, including phytosterols, carotenoids, and peel waxes. The washed residue (3 g) was dissolved in 200 mL toluene and loaded onto a 200 g silica gel column, where linear gradients of hexane and ethyl acetate were run to isolate the tangeretin from other peel oil flavonoids, primarily nobiletin. With repeated passes over silica gel columns, nearly pure (>97%) tangeretin was obtained as a white solid [24].

### 2.4. Preparation of Tangeretin Microcapsules

Solutions of 3% (*w*/*v*) sodium alginate and 3% (*w*/*v*) pectin were prepared by slowly adding 1.5 g of sodium alginate or 1.5 g of pectin into 50 mL of DI water. These suspensions were stirred for 16 h at 700 rpm to fully hydrate the biopolymer molecules. The pectin solution was then slowly added in to the sodium alginate solution with stirring to obtain a 100 mL 3% pectin/sodium alginate solution. Tangeretin-in-bergamot oil solutions were prepared by adding 2 mg (low tangeretin) or 20 mg (high tangeretin) of tangeretin into 1 g of bergamot oil. To improve the solubility, the latter was warmed up to 60 °C. Bergamot oil without tangeretin was used as control. The emulsifier Tween^®^ 80 (5 mL) was combined with the tangeretin-in-bergamot oil solution or control, and then the total volume was slowly added to the pectin/sodium alginate suspension. Emulsification was conducted using a homogenizer (Bio-Den Series Pro200; PRO Scientific, Oxford, CT, USA) at 12,000 rpm for 5 min. The emulsions were spray dried with a spray dryer (Model B-290; BUCHI, New Castle, DE, USA). The spray drier was fit with a two-fluid nozzle consisting of a 1.5 mm diameter nozzle cap with a 0.7 mm diameter nozzle tip hole. The inlet air temperature, feed rate, aspiration, and air flow rate were set at 100 °C, 7 mL min^−1^, 100%, and 26 L min^−1^, respectively. For high-tangeretin microcapsules, both oil and aqueous solutions, before and after mixture, were kept warm (>60 °C) throughout the homogenizing and spray-drying processes to maintain the complete solubility of tangeretin in the bergamot oil [3].

### 2.5. Morphology and Particle Size Distribution of the Microcapsules

The outer topography of the microcapsules was inspected by a scanning electron microscope (SEM, Hitachi S4800; Hitachi High Technologies America, Inc., Pleasanton, CA, USA). Samples were analyzed after being attached to the SEM stubs with an accelerating voltage of 5.0 kV and a magnification of 2000X. In order to study the inner structure, a small amount of the microcapsules were sprinkled onto a drop of clear nail varnish (Sally Hansen Advanced Hard as Nails; Coty US LLC, Morris Plains, NJ, USA) covering the top surface of a 15-mm aluminum SEM specimen mount pin stub (Cat. #75230; Electron Microscopy Sciences, Hatfield, PA, USA). The sample was kept overnight in a drying oven at 57 °C. While viewed under a dissection scope, the polish was cut into sections using a single-edged razor blade (GEM Scientific; American Safety Razor Co., Staunton, VA, USA). Each section was then turned up on edge while still on the mounting stub. The sample was sputter-coated with gold/palladium for 1.5 min with a 10 mV plasma current (HUMMER^®^ 6.2; Annatech, Union City, CA, USA) and viewed as described above with a magnification of 8000X. Images were captured and measured using Quartz PCI imaging software (V6E 4G1; Quartz Imaging Corp., Vancouver, BC, Canada).

The size and volume mean diameter (VMD) of the particles were evaluated using a particle analyzer (HELOS (H3863) and RODOS/L; Sympatec, Indianapolis, IN, USA) with a 0.5 bar pressure and 20 mbar vacuum. The distribution and cumulative distribution curves were obtained using Microsoft Excel (ver. 2016; Microsoft, Redmond, VA, USA).

### 2.6. Physical Properties of the Microcapsule Powder

#### 2.6.1. Moisture Content

The moisture content of the microcapsule powder was measured according to a previous study [25]. Briefly, the powder was dried in a glass chamber at 100 °C until a stable weight was obtained, and the moisture content was expressed as percentage of the initial weight which was lost.

#### 2.6.2. Bulk Density

The bulk density of the microcapsule power was evaluated following a previous study [26]. Briefly, bulk density was determined by the volume of 1 g of the microcapsule powder in a 5 mL cylinder after being tapped 50 times manually on a flat surface.

#### 2.6.3. Dissolution Time

The time required for complete dissolution of the microcapsule powder was calculated using a slightly modified procedure originally described in a previous study [25]. Briefly, 0.5 g of the microcapsule powder was added to 10 mL of DI water and stirred at 700 rpm. The time necessary for the powder to completely dissolve was assigned as the dissolution time. 

#### 2.6.4. Hygroscopicity

Hygroscopicity was calculated by distributing a 1 g sample of each microcapsule powder evenly on a 9 cm diameter dry plate and then the hygroscopicity value was assigned based on the increase in weight per 100 g of powder at the end of a 90 min storage time in a closed box containing a sodium sulfate saturated solution at 23 °C and 76% relative humidity [26].

### 2.7. Retention Efficiency and Encapsulation Efficiency of Tangeretin in the Microcapsule Powder

#### 2.7.1. Total Tangeretin of the Microcapsule Powder

The microcapsule powder was dissolved in methylene dichloride at a concentration of 100 mg per 10 mL by stirring for 30 min at 700 rpm, followed by 1 h ultrasonic extraction using an ultrasonicator (Model 2800, Branson Ultrasonics, Danbury, CT, USA) which was set to a frequency of 20 kHz and at 70% amplitude. Subsequently, the solvent was filtered through a 0.2 μm syringe filter. The suspension was dried under nitrogen, and then dissolved in 1 mL of methanol. Mass spectral (MS) analysis of the tangeretin was performed using a single-quadrupole mass spectrometer equipped with a High-Performance Liquid Chromatography (HPLC) pump with a detector (Waters ZQ, Waters 2695 Alliance, and Waters 996; all Waters Corp., Milford, MA, USA). The specific parameters for the MS and HPLC settings were described in a previous work [27]. Compound separation was achieved with a Waters XBridge C8 column (4.6 × 150 mm id) using linear gradients of solvent A, 0.5% aqueous formic acid, and solvent B, acetonitrile. The linear gradients were as follows: 0 min, 99% A, 1% B; 10 min, 95% A, 5% B; 20 min, 80% A, 20% B; 40 min, 70% A, 30% B; 48 min, 25% A, 75% B; 53 min, 25% A, 75% B; 60 min, 99% A, 1% B; 80 min, 99% A, 1% B; at a constant flow rate of 0.3 mL min^−1^. HPLC eluent was split between the photodiode array detector and the mass ZQ detector in a 10:1 ratio. UV spectra were monitored between 240 and 400 nm, and chromatograms were monitored at 280 and 330 nm. Data handling was performed with MassLynx software ver. 4.1 (Micromass, Division of Waters Corp., Beverly, MA, USA). MS parameters were as follows: ionization mode, positive electrospray; capillary voltage, 3.0 kV; extractor voltage, 5 V; source temperature, 100 °C; desolvation temperature, 225 °C; desolvation nitrogen gas flow, 465 L h^−1^; cone nitrogen gas flow, 70 L h^−1^; scan range, *m*/*z* 100–900; scan rate, 1 scan s^−1^; cone voltages, 20 and 40 eV. The identification of tangeretin was performed by absorbance and mass spectrometry, and by comparison of the retention time of samples and an authentic standard. The evaluation of the total tangeretin concentration was accomplished using a standard curve developed by measuring serial dilutions of tangeretin dissolved in methylene dichloride. 

#### 2.7.2. Surface Tangeretin of the Microcapsule Powder

The amount of tangeretin on the surface of the microcapsules was calculated by rinsing 0.25 g powder with 10 mL methylene dichloride and stirring at 700 rpm for 5 min. The suspension was dried under nitrogen, and then dissolved in 1 mL of methanol. MS analysis of the surface tangeretin was performed using a single-quadrupole mass spectrometer equipped with a HPLC pump with a detector (Waters ZQ, Waters 2695 Alliance, and Waters 996; all Waters Corp., Milford, MA, USA). The specific parameters for the MS and HPLC settings were the same as with the total tangeretin assessment described in Section 2.7.1. A standard curve for tangeretin concentration was created by dissolving pure tangeretin in methylene dichloride at a series of concentrations. The surface tangeretin concentration of the microcapsules was determined by referencing the standard curve. The percentage of total tangeretin retained during the drying process defines the retention efficiency value, while the percentage of retained tangeretin that is successfully entrapped into the microcapsules defines the encapsulation efficiency value [28]. The retention efficiency of tangeretin was calculated as the percentage of total tangeretin added in the formulation that was subsequently found in the microcapsule powder, both as encapsulated tangeretin and tangeretin present on the surface of microcapsules; the encapsulation efficiency of tangeretin was calculated as the percentage of the total tangeretin found in the microcapsule powder that was actually encapsulated (total retained tangeretin, excluding surface tangeretin). The retention efficiency and encapsulation efficiency of tangeretin in the microcapsule powder were calculated by the following Equations (1) and (2), respectively.

*Retention efficienc*y (%) = (*Total tangeretin of the powder/Total tangeretin added*) × 100 (1)

*Encapsulation efficiency* (%) = ((*Total tangeretin of the powder − surface tangeretin*)/*Total tangeretin of the powder*) × 100 (2)

### 2.8. Statistical Analysis

Results were evaluated using analysis of variance (ANOVA) and subsequently Tukey’s HSD test using JMP (version 11.2.0; SAS Institute, Cary, NC, USA). All of the experiments were repeated three times. The significance was defined at *p* < 0.05.

## 3. Results and Discussion

### 3.1. Morphology and Particle Size Distribution of the Microcapsules

**Figure 1:**
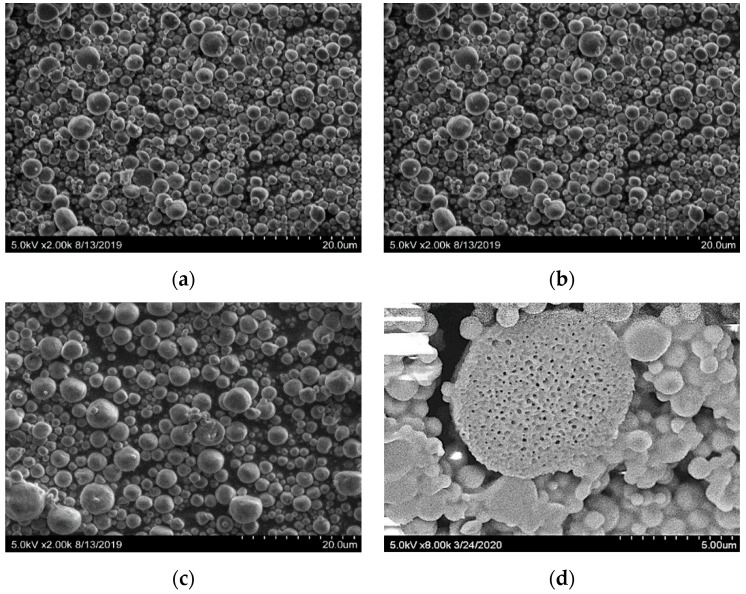
Scanning electronic microscopy (SEM) images of tangeretin in citrus pectin and sodium alginate matrix microcapsules. (**a**): Control (no tangeretin); (**b**): low tangeretin; (**c**): high tangeretin; (**d**): inner structure of the investigated microcapsules.

The SEM photomicrographs of microcapsules showed a smooth, spherical, and complete surface (Figure 1a–c). The individual emulsion particles were seen to be spherical when viewed through an electron microscope, which is an essential criterion for the food industry [29]. This is important because a more uniform spherical shape has been confirmed to correspond to reduced gas permeability as well as improved retention and protection of the encapsulated material [30,31]. The structural features of spray-dried microcapsules are affected by the combined influence of the composition of the emulsion, physicochemical properties of the emulsion and its constituents, and by the atomization and drying conditions of capsule formation [32]. In this study, all of the various emulsions were spray dried using the same inlet air temperature, air flow rate, feed rate, and aspiration conditions. Spherical microcapsules were obtained for all of the produced emulsion types (Figure 1a–c). The dry microcapsules exhibited smooth outer surfaces with a very limited incidence of surface irregularity (Figure 1a–c). Structural features of the outer topography of the investigated microcapsules were similar to those that were reported for spray-dried microcapsules prepared with various ratio blends of wheat protein isolate and lactose [32]. There are many types of microcapsules, including simple microcapsules, matrix (microsphere) microcapsules, irregular microcapsules, multicore microcapsules, multiwall microcapsules, and assemblies of microcapsules. A close examination of the inner structure of the microcapsules revealed matrix (microsphere) microcapsules. Images of these matrix (microsphere) microcapsules were obtained, and are shown in Figure 1d. The presence of these matrix (microsphere) microcapsules indicates a typical matrix inner structure of oil-containing spray-dried microcapsules and was seen in all microcapsule formulations. 

Additionally, central voids were visible and the active component had been successfully encapsulated. The small droplets dispersed in the pectin/sodium alginate wall matrix had an average diameter of 198.33 nm. Formation of central voids in microcapsules is related to the expansion of the particles during the latter stages of the drying process [33]. These matrix-type microcapsules can release tangeretin in a controlled manner. This result is similar to what has been observed for microcapsules prepared with wall systems containing carbohydrates and proteins [34].

**Figure 2:**
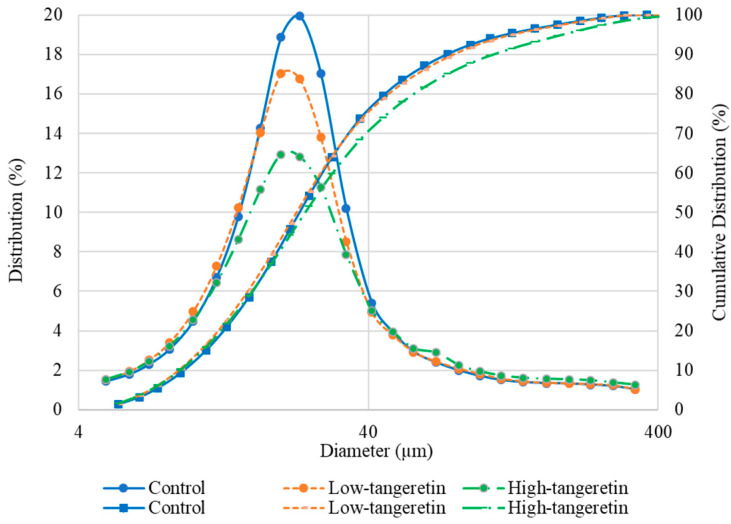
Particle size distribution of microcapsules with tangeretin encapsulated in citrus pectin and sodium alginate matrix.

The average individual particle size of a powder is a factor that impacts other features related to storage, handling, and transportation, specifically, powder solubility, the flow-ability of the powder, the powder’s capacity for rehydration, and the powder’s angle of repose [30]. Particle size will also affect the stability of encapsulated ingredients, which are sensitive to environmental factors such as high and low temperature extremes, ambient light, and relative humidity [30]. The results of this study revealed that the mode of the distribution curve varied between 22.70 and 24.31 µm (Figure 2). The volume mean diameters (VMDs) of microcapsules from control, low-tangeretin, and high-tangeretin samples were 36.69, 37.33, and 47.17 µm, respectively. Comparable results have been found in alginate/HPMC hydrogel beads [35]. Particle sizes varying from 2 to 120 µm have been reported for microencapsulated fish oil in a casein-pectin biocomposite shells produced via a spray-drying process [19].

### 3.2. Physical Properties of the Microcapsule Powder

The concentration of tangeretin did not cause a significant difference in the physical properties of the microcapsule powder. The powder possessed a moisture content between 4.47% and 4.70% (Table 1). These moisture content levels meet the standards for industrial use, considering that a 5% or lower total moisture content is deemed optimal to sufficiently reduce lipid oxidation and microbial contamination [14], and therefore the moisture content of the microcapsules produced in this study should not unfavorably alter the powder’s ability to be reliably stored for an extended period of time [13]. A similar moisture content was achieved in earlier studies [8,14,36,37]. The microcapsules with carvacrol encapsulated in a pectin/sodium alginate matrix showed a range of 4.2–6.3% moisture content which decreased with an increasing spray-drying outlet temperature [8]. Pectin alginate-encapsulated α-tocopherol microcapsules showed moisture content in a range between 4.29% and 4.73% [13]. 

The bulk density of powders is affected by chemical composition, particle size, and moisture content as well as by processing and storage conditions [38]. In principle, density increases as volume decreases when mass is constant. The bulk density of the microcapsule powder was 0.31 g cm^−3^ (Table 1). High bulk density values translate to smaller container requirements and, therefore, more convenient storage. Furthermore, a higher bulk density value indicates a reduced amount of air, resulting in reduced vulnerability to oxidation [25]. Taking these considerations into account, the bulk density of the produced powder suggests that it would not be prohibitively inconvenient for use in the food industry [14]. In our previous research, a bulk density ranging from 0.28 to 0.35 g cm^−3^ was obtained using pectin and sodium alginate as the wall material [8]. The bulk density of spray-dried powders containing avocado oil encapsulated in whey protein and maltodextrin was between 0.25 and 0.28 g cm^−3^ and increased gradually as the concentration of maltodextrin increased [38].

Tangeretin is a flavonoid that is soluble in either methanol or ethyl acetate, but insoluble in water. The spray-drying encapsulation process improved the water solubility of the microcapsules. The ability of spray-dried powders to mix with water or other powders is one of the most important physical properties related to reconstitution with water or dry blend formulation [38]. Dissolution time is the affinity with which a liquid maintains contact with a solid, and is dictated by a balance between intermolecular adhesion and cohesion [39]. Dissolution time is broadly defined as the ability to rehydrate in water. In other words, and more specific to this application, dissolution time is the ability of a bulk microcapsule powder to absorb water [14]. The dissolution time of the microcapsule powder was predominately dictated by the matrix structure and the emulsifier concentration [14]. It also depended upon powder particle size, bulk density, porosity, surface charge, surface area, presence of amphiphthic substances, and the surface activity of powders [38]. The dissolution time of the microcapsule powder was approximately 5 min (Table 1). A more condensed physical structure resulted in a powder with a higher bulk density, while increasingly hydrophilic wall materials lead to longer dissolution times [38].

The microcapsule powder hygroscopicity after 90 min at 76% RH and 23 °C was between 2.70 and 3.03 g of water per 100 g of the microcapsule powder (Table 1). Particles produced using a spray-drying process have the propensity to readily absorb water from the surrounding air, at which point the powder will become pasty and form a sticky mass [26]. The high hygroscopicity value for microcapsule powder indicates an area requiring additional investigation into the effect of water content on stability and shelf life.

### 3.3. Retention Efficiency and Encapsulation Efficiency of Tangeretin in the Microcapsule Powder

The retention efficiencies of citrus pectin/sodium alginate-encapsulated tangeretin were 71.05% and 98.92% when the concentrations of tangeretin were 0.2% and 2% (*w*/*w*) of bergamot oil, respectively (Table 2). A higher retention efficiency of tangeretin was achieved when the concentration of tangeretin was increased. This was probably due to the elevated temperature (60 °C) of the high-tangeretin emulsion during the homogenizing and spray-drying processes. Yang et al. [3] found that the solubility of tangeretin in bergamot oil was significantly increased after heat treatment. Our previous research showed that the retention efficiency of carvacrol was 88.69% in the same pectin/sodium alginate matrix under the same spray-drying conditions, and that a higher inlet air temperature resulted in a lower retention efficiency [8]. 

The encapsulation efficiencies of tangeretin in the microcapsule powder using bergamot oil as a carrier were 75.64% and 55.51% when the concentrations of tangeretin were 0.2% and 2% (*w*/*w*) of bergamot oil, respectively (Table 2). Pectin is well suited both as a coating and a gelling material. The alginate–pectin mixture has been shown to provide a protective effect by forming a strong gel matrix under acidic conditions [40]. Therefore, this mixture has been successfully used for the encapsulation of several nutrients and drugs, such as vitamins [13], essential oils [41], and proteins [42]. The encapsulation efficiency of α-tocopherol in microencapsules produced with wall materials comprising 2.0% (*w*/*v*) pectin and 1.5% (*w*/*v*) sodium alginate was 52.91% [13]. Our previous research showed that the pectin and sodium alginate matrix could successfully encapsulate more than 90% of the provided carvacrol [8]. 

Particles formed from microemulsions with larger droplet sizes had a higher amount of oil on their surfaces, which is assumed to be a result of the droplets rupturing during atomization [31]. The average size of the individual particles of microcapsule powder is predicted to be one of the most important factors impacting encapsulation efficiency, as highly concentrated tangeretin showed larger particle sizes with a higher VMD when encapsulated in a citrus pectin and sodium alginate matrix. This indicates that more tangeretin adhered to the exterior of the microcapsule as the surface area increased, which resulted in an efficiency reduction [43]. A related study also indicated that decreasing the particle size improved the encapsulation efficiency of active compounds, such as flavors and essential oils [44]. 

## 4. Conclusions

Our results indicate that our tested spray-drying conditions could successfully encapsulate different concentrations of tangeretin into a citrus pectin/sodium alginate matrix using bergamot oil as a carrier. The SEM images and particle size distribution results indicated that the produced matrix particles were smooth and spherical, with a volume mean diameter of approximately 40 µm. The encapsulation of tangeretin was accomplished, achieving promising physical properties with comparatively high retention and encapsulation efficiencies. In conclusion, this procedure for producing a small encapsulating structure constructed of citrus pectin and sodium alginate could be used for the industrial encapsulation of tangeretin and other functional substances. The produced microcapsules could be employed in a wide range of food and pharmaceutical industries, including in dairy products, processed foods, and nutraceutical supplements based on the properties of the encapsulated ingredient and the needs of the consumer. 

## Figures and Tables

**Table 1:** Physical properties of tangeretin in citrus pectin and sodium alginate matrix microcapsules.

Samples	Moisture Content (%)	Bulk Density (g cm^−3^)	Dissolution Time (min)	Hygroscopicity (g H_2_O 100 g^−1^)
Control (no tangeretin)	4.47 ± 0.75	0.31 ± 0.01	5.03 ± 0.12	2.70 ± 0.30
Low tangeretin	4.63 ± 0.12	0.31 ± 0.01	4.97 ± 0.06	3.03 ± 0.31
High tangeretin	4.70 ± 0.20	0.31 ± 0.01	4.93 ± 0.32	2.80 ± 0.36

**Table 2:** Retention efficiency and encapsulation efficiency of tangeretin in citrus pectin and sodium alginate matrix microcapsules.

Samples	Retention Efficiency (%)	Encapsulation Efficiency (%)
Low tangeretin	71.05 ^b^	75.64 ^a^
High tangeretin	98.92 ^a^	55.51 ^b^

Different letters in same column indicate significant differences by the Tukey’s HSD test (*p* < 0.05).
